# Emergent Dynamics of Fairness in the Spatial Coevolution of Proposer and Responder Species in the Ultimatum Game

**DOI:** 10.1371/journal.pone.0116901

**Published:** 2015-01-27

**Authors:** Reiji Suzuki, Tomoko Okamoto, Takaya Arita

**Affiliations:** 1 Graduate School of Information Science, Nagoya University, Nagoya, Aichi, Japan; 2 Department of Forest Entomology, Forestry and Forest Products Research Institute, Tsukuba, Ibaraki, Japan; Université de Lausanne, SWITZERLAND

## Abstract

While spatially local interactions are ubiquitous between coevolving species sharing recourses (e.g., plant-insect interactions), their effects on such coevolution processes of strategies involving the share of a resource are still not clearly understood. We construct a two-dimensional spatial model of the coevolution of the proposer and responder species in the ultimatum game (UG), in which a pair of proposer and responder individuals at each site plays the UG. We investigate the effects of the locality of interactions and the intensity of selection on the emergence of fairness between these species. We show that the lower intensity of selection favors fair strategies in general, and there are no significant differences in the evolution of fairness between the cases with local and global interactions when the intensity of selection is low. However, as the intensity of selection becomes higher, the spatially local interactions contribute to the evolution of fairer strategies more than the global interactions, even though fair strategies become more difficult to evolve. This positive effect of spatial interactions is expected to be due to the mutual benefit of fairness for both proposer and responder species in future generations, which brings about a dynamic evolution process of fairness.

## Introduction

The evolutionary dynamics of strategies for the ultimatum game (UG) has been extensively discussed in the context of emergence of fairness since it was proposed by Guth et al. [1]. There are two players (proposer and responder) in this game, and they have to decide how to divide a resource. The proposer suggests an offer and the responder decides whether to accept it or not. If it is accepted, the resource is distributed between the players, which means that the responder receives the offer and the proposer receives the remaining share. However, both players get nothing if it is not accepted. The expected behavior of the rational responder is to accept any amount of the offer because rejecting the offer brings about no benefit. Under the assumption of this rational behavior of the responder, it is clear that the rational strategy of the proposer is to offer the minimal amount of the resource. However, it is well known that humans tend to make fairer decisions offering more amount of resources and rejecting smaller amount, which are expected to be due to both genetic [2] and cultural [3] factors. Many studies have been conducted in order to find mechanisms that enable fairness to emerge in the UG and various versions of UG [4, 5] have also been studied recently.

Especially, spatial or local interactions have been recognized as one of the key mechanisms for evolution of fairness, since Page et al.’s pioneering work [6]. They assumed that individuals who have both proposer and responder strategies are distributed in one or two dimensional grid space, and the population evolves according to local reproductions based on the fitness obtained from the UG played among neighboring individuals. They clarified the condition in which the cluster of fair individuals can grow in the sea of less fair individuals under the assumption that the individual with the best fitness always invades into its neighboring sites. This process was possible because such fair individuals are able to make others’ fitness small by rejecting the offer from less fair individuals and forcing them to accept their offer which assures their benefit. This means that the combination of the proposer and responder strategies is important for such an invasion process. Iranzo et al. further investigated in more detail by focusing on types of reproduction rules, effects of noises and fixed topologies of interactions [7]. It was reported that the spatial locality can facilitate the evolution of fairness in various conditions. Recently, Gao et al. discussed the evolution of strategies for the UG on adaptive networks in which a random link between two individuals is rewired if the offer from a proposer is not accepted [8]. The results showed that the promotion of fairness caused by rewiring process was limited due to the emergence of isolated individuals, and that the small average degree led to a fair society if the rewiring probability was small. Szolnoki et al. also showed that rich patterns of strategies (e.g., spiral) emerged in the two-dimensional spatial UG with empathetic and discrete strategies [9]. These studies clarified that the spatial or topological properties of interactions can have significant effects on the emergence of fairness in the UG.

In the paper reported here, we focus on another type of spatial locality in which two different species play the UG as a proposer and a responder, respectively. We can see such situations in plant-insect relationships, for example. Recently, it has been reported that there exists species-specific pollination mutualism between *Glochidion* trees and *Epicephala* moths [10, 11]. In this obligate pollination-seed consumption mutualism, the moths deliver the pollination service by actively discriminating between male and female flowers using their different scents [12] and lay eggs on flowers. The hatched larvae consume a subset of the resulting seeds and the remaining seeds contribute to plant reproduction. It should be noted that there is a selective abortion mechanism of flowers containing excessive eggs [13]. This means that the plants correspond to fair responders in the UG. In this situation, the resulting seeds can be interpreted as the shared resource in the UG. The number of unconsumed seeds by larvae can be interpreted as the strategy of the proposer, and its threshold number for aborting the whole flower can also be interpreted as the strategy of the responder. The moths have chances to lay eggs on multiple female flowers. However, they cannot make sure whether the flowers with their eggs have been aborted or not because they die before the plants abort the flowers. Thus, there are no feedback mechanisms between pollination events. In this sense, an interaction process between an insect and a flower can be regarded as one-shot UG in this context. Such a relationship is also observed in plant-bacteria mutualism for nitrogen fixation [14]. These studies imply that this type of mutual relationship that corresponds to the UG is widespread in real biological systems, especially in plant-non-plant interactions in which the spatial locality is supposed to affect them significantly. Rand et al. showed analytically that, there is no qualitative difference in the two population case of proposers and responders discussed above and a single population case in which an individual can be both proposer and responder, under the assumption of the weak selection limit with global interactions [15]. However, as far as we know, effects of the spatial locality on the coevolution process of proposer and responder species has not got so much attention in the context of the evolutionary UG.

Our purpose is to give a valuable insight into the spatial coevolution of the proposer and responder species in the UG. For this purpose, we construct a two-dimensional spatial model of the coevolution of proposer and responder species in which a pair of proposer and responder individuals at each site plays the UG. We investigate the effects of the spatially local interactions on the emergence of fair relationship between these species. We also focus on the effects of the intensity of selection on coevolution processes. Rand et al. recently reported that the intensity of selection was another important factor for the emergence of fairness, showing that fair behaviors were facilitated when the intensity of selection was relatively low [15]. In this study, we show the lower intensity of selection favors fair strategies in general, and there are no significant differences in the evolution of fairness between the cases with local and global interactions when the intensity of selection is low. However, as the intensity of selection becomes higher, the spatially local interactions contribute to the evolution of fairer strategies than the global interactions despite that the fair strategies become more difficult to evolve. We discuss the dynamic evolution process that brings about the emergence of such fair strategies in the condition of higher intensity of selection.

## Method

**Fig 1:**
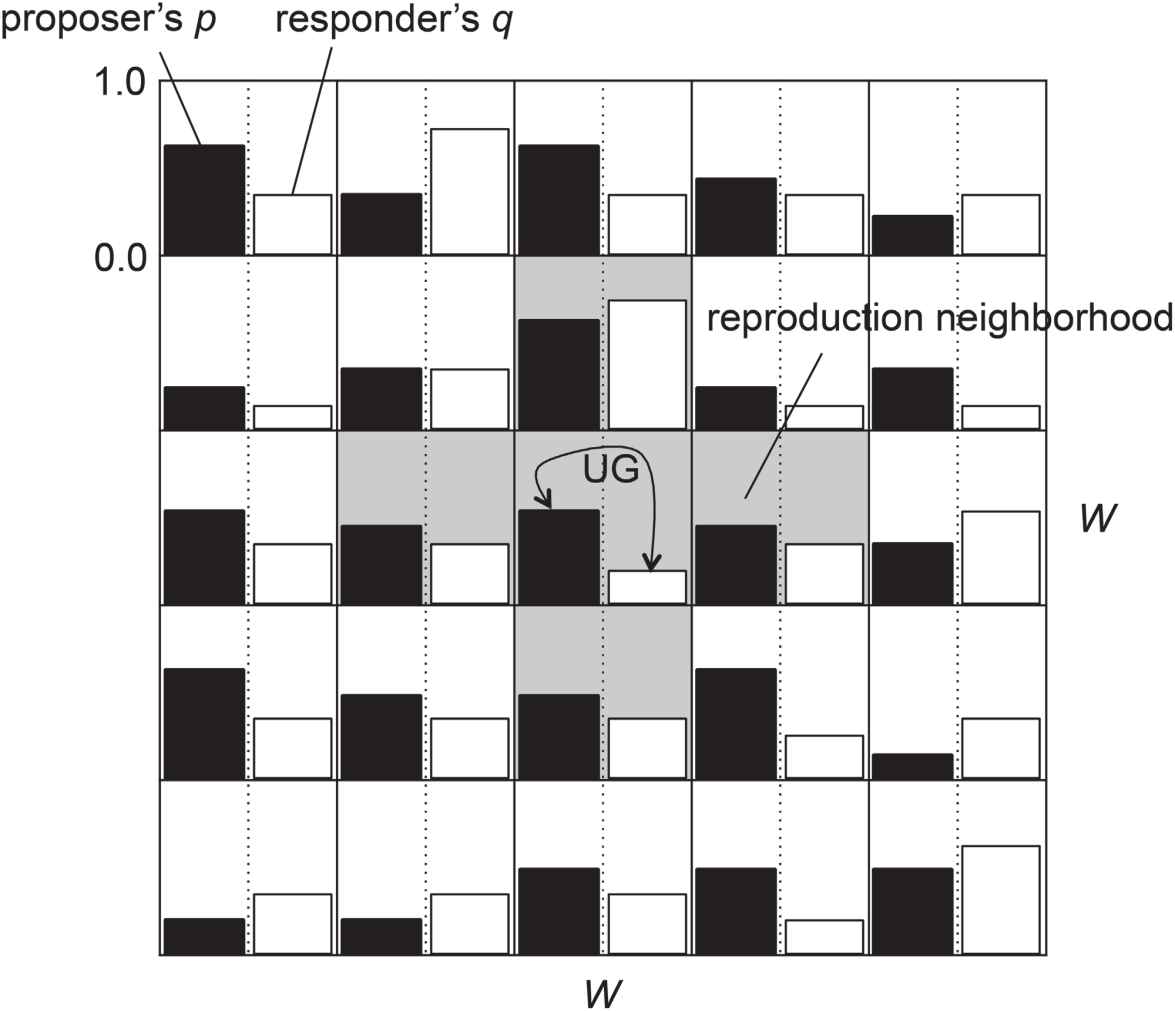
A two-dimensional model of the coevolution of proposer and responder species in the ultimatum game (UG). A pair of proposer and responder individuals at each site play the UG. The populations of both species evolve locally.

We construct a two-dimensional spatial model of the coevolution of proposer and responder species. There are toroidal *W* × *W* lattice sites and each site contains two individuals. One is an individual of the proposer species and the other is an individual of the responder species, as shown in Fig. 1. Each proposer has a genetically determined proportion of the resource *p* ∈ [0, 1] to which it proposes an individual of the responder species. Each responder has a genetically determined threshold *q* ∈ [0, 1]. It represents the minimum proportion of the offer from the responder species that it accepts.

A pair of a proposer with its offer *p* and a responder with its threshold *q* receives the following payoff *f*_*p*_ and *f*_*q*_, which will also be their fitness (with no effects from the previous generation), respectively:

$$\left(f_{p},f_{q}\right)=\left\{\begin{array}{cc} \left(1-p,p\right) & \text{if}\;p\geq q,\\ \left(0,0\right) & \text{otherwise.} \end{array}\right.$$ (1)

In this study, we assume the two different cases of interactions between the proposer and responder species. In the case of spatially local interactions, a pair of individuals in each site play an ultimatum game in each generation. Note that we adopted the two-dimensional grid as a typical example representing the spatial locality, which is expected to have a significant effect on the coevolution of strategies due to its regularity. Then, the evolution process of each species is conduced locally according to the scaled fitness *e* ^*α*×*f*^, where *α* represents the intensity of selection and the original fitness *f*. Specifically, the probability with which a neighboring individual *j* around the site *i* is selected as a parent of the offspring at the focal site *i* (*p*_*i*, *j*_) is as follows:

$$p_{i,j}=\frac{e^{\alpha f_{j}}}{\sum_{k\in N_{i}}e^{\alpha f_{k}}},$$ (2)

where *N*_*k*_ is the set of the five individuals at the Neumann neighborhood around the site *k* including the focal site itself. *α* is the parameter which determines the intensity of selection. As *α* becomes larger, the best neighbor tends to be selected more often, and the evolution process will be equivalent to the case in which the best neighbor invades into the focal site when *α* is infinitely large.

As *α* becomes smaller, the selection process becomes more stochastic and less adaptive individuals tend to be selected more often. If *α* is close enough to 0, the selection process becomes almost neutral (no selection pressure). This update process of each genotype occurs at the same time in the population of each species, independently. We also assumed that a mutation can occur with a small probability *p*_*m*_ for each offspring. When it occurs, a random value from the uniform distribution of [0, 1] is assigned to its genotypic value of *p* or *q*.

In the case of global interactions as control experiments, we add a random movement of proposer species before interactions, which can be interpreted as a migration of insects in an obligate pollination mutualism: All proposers and responders are on the two-dimensional grid as assumed in the case of local interactions. However, each proposer migrates to a randomly chosen site from the whole sites without duplication, and then an ultimatum game is played between a new pair of individuals in each site. The reproduction process occurs according to the new spatial location after the migration, that is, in each site, one of the five nearest neighbors including the focal cite is selected as a parent of the offspring. Thus, this case corresponds to the control experiment with no spatially local interactions (but spatially local reproductions in the sense above). Another reason for adopting the local reproduction in the control experiments is that the global reproduction based on a selection of offspring from the whole population makes evolution processes too disordered to be compared reasonably to that in the original experiments especially when the intensity of selection is high. This is expected to be due to the fact that an individual who obtains the highest fitness by chance immediately dominates the whole population.

## Results and Discussion

### Basic analyses

**Fig 2:**
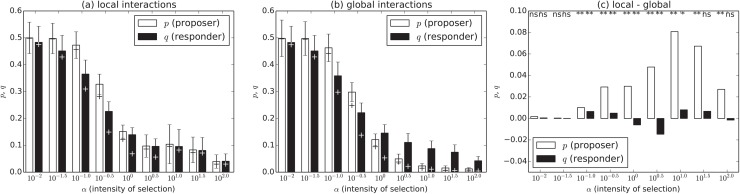
The average proposer’s *p* and responder’s *q* with various conditions of the intensity of selection *α* in the cases with local (left) and global (center) interactions. Each cross also indicates the median of the corresponding value. The difference in these values between these cases and the statistical significance (t-test) of the difference are also shown (right). The P-values, as determined by a two-tailed t-test, are indicated by double asterisk (*P* < 0.001), asterisk (*P* < 0.05) and ns (not significant).

We conducted evolutionary experiments for 20,000 generations using the parameter settings as follows: *W* = 25, *p*_*m*_ = 0.015. As for the intensity of selection *α*, we used 10^−2.0^, 10^−1.5^, ⋯, and 10^2.0^. We assumed that the initial values of *p* and *q* for each individual are extracted from a uniform distribution from 0 to 1. Fig. 2 shows the average proposer’s *p* and responder’s *q* over the last 15,000 generations with various conditions of the intensity of selection *α* in the cases with local (a) and global (b) interactions. Each cross also indicate the median of the corresponding value, and each error bar represents the standard deviation of “the average *p* and *q* in each generation” over all the generations. The difference in these values between these cases and the statistical significance (t-test) of the difference are also shown in (c). The values are averaged over 30 trials.

In Fig. 2 (a) and (b), we see that the average *p* was slightly higher than the average *q* in general, meaning that the two population coevolved so that the responder tended to accept the proposal in general. Although the average *q* was larger than the average *p* in the cases with the large *α* (≥ 10^0.0^) and global interactions, we confirmed that the population of the responder species was basically dominated by the individuals with the smaller *q* than *p* of their opponent but a few mutant individuals with large *q* coexisted, which is the reason for the larger average *q* than the average *p*. Thus, the median of *p* was larger than that of *q* in these conditions.

We also see that both average *p* and *q* tended to be smaller as *α* increased in both cases of local and global interactions in general, meaning that the lower intensity of selection contributed to the evolution of fair strategies.

However, as shown in Fig. 2 (c), we see that the average proposer’s *p* in the cases with local interactions tended to be higher significantly than the corresponding values in the cases with global interactions as the intensity of selection became higher (*α* ≥ 10^−1.0^). Considering that the median of *q* with local interactions was also higher than that with global interactions as shown in Fig. 2 (a) and (b), most responders in the locally interacting population had higher *q*. On the other hand, there were no statistically significant differences in the results between local and global interactions when the intensity of selection was low (*α* ≤ 10^−1.5^).

This means that the spatially local interactions facilitated the evolution of fair strategies especially when the intensity of selection was high.

### Distribution of the average strategies

**Fig 3:**
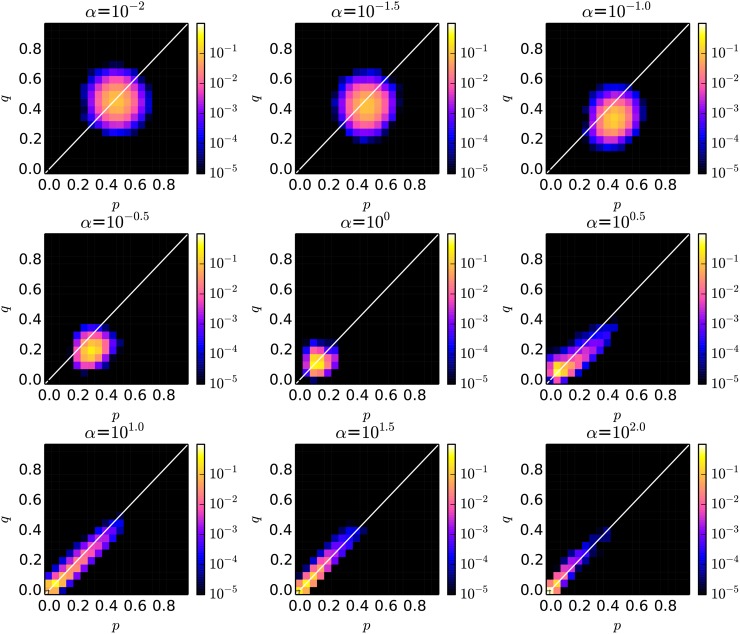
The probability distribution of *p* and *q* with various conditions of the intensity of selection *α* in the case with local interactions between proposer and responder species.

**Fig 4:**
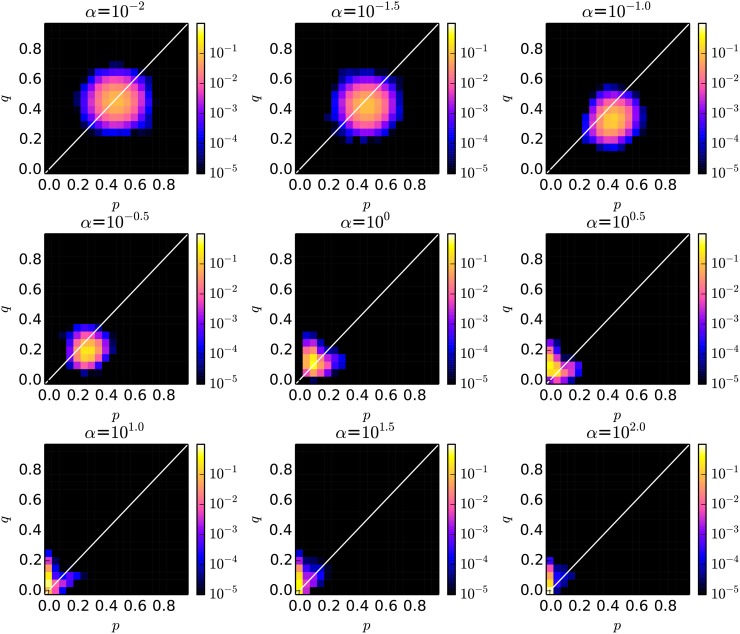
The probability distribution of *p* and *q* with various conditions of the intensity of selection *α* in the case with global interactions between proposer and responder species.

Next, we focus on the distribution of the pair of the average *p* and *q* through generations discussed above. Figs. 3 and 4 show the two dimensional histogram of “the pair of the average *p* (x-axis) and *q* (y-axis) in each generation” over the last 15,000 generations in the cases with the local and global interactions, respectively. Both values were split into bins, with each bin represents an interval of 0.05. We see that the distribution tended to be significantly different as *α* became large.

More specifically, when *α* was small (*α* ≤ 10^−1.5^), both *p* and *q* were widely distributed around 0.5 in both cases of interactions while keeping the average *p* slightly higher than *q*. The reason for this high fairness is related to the one discussed in [15]. That is, because there existed large variations and fluctuations in the thresholds of the responder species in the case with the lower intensity selection, the proposer species were necessary to increase the amount of proposal so that its offer can be accepted by a lot of responders with the higher threshold (than the average). The distribution of the average *p* and *q* moved toward the smaller values keeping *p* > *q* as *α* increased.

At the same time, when *α* was large (*α* ≥ 10^0.5^), we see that their evolution process became more dynamic only in the cases with local interactions. While both *p* and *q* tended to be the smallest values around 0.05, they sometimes evolved to be larger values simultaneously as we see their long-tailed distribution between (0.05, 0.05) and (0.5, 0.5). We did not see such a process in the case with global interactions, and *p* and *q* tended to converge to the very small values. Thus, this correlated evolution process is expected to be the reason why fairer relationship was established in the cases with the local interactions compared with the cases with the global interactions.

On the contrary, in the cases with the global interactions, we also see that the average *q* tended to be higher than *p* when *α* was high. As explained before, this higher average *q* is due to the coexistence of dominant individuals with smaller *q* and a few mutant individuals with much larger *q*. In this situation, the amount of obtained resource by the responder is almost 0 due to the extremely small *p* of the proposer species. The scaled fitness of the accepting responder is almost 1.0. On the other hand, the scaled fitness of the non-accepting responder is *e* ^0.0^ = 1.0, which is not zero. Thus, the net difference in the scaled fitness between the mutant responders and accepting responders becomes small. This sometimes allows mutant individuals with randomly assigned *p*s to survive in the population for a few generations, which increases the average *p* although they do not have ability to dominate the whole population.

### Evolutionary dynamics of fairness under the high intensity of selection

**Fig 5:**
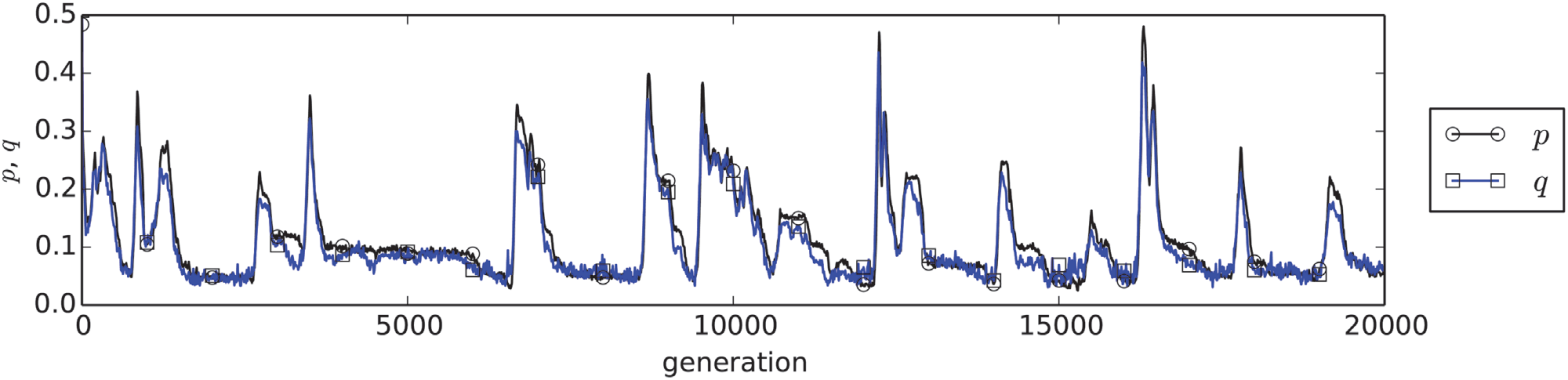
An example evolution process of *p* and *q* when *α* = 10.0 in the case with local interactions between proposer and responder species.

**Fig 6:**
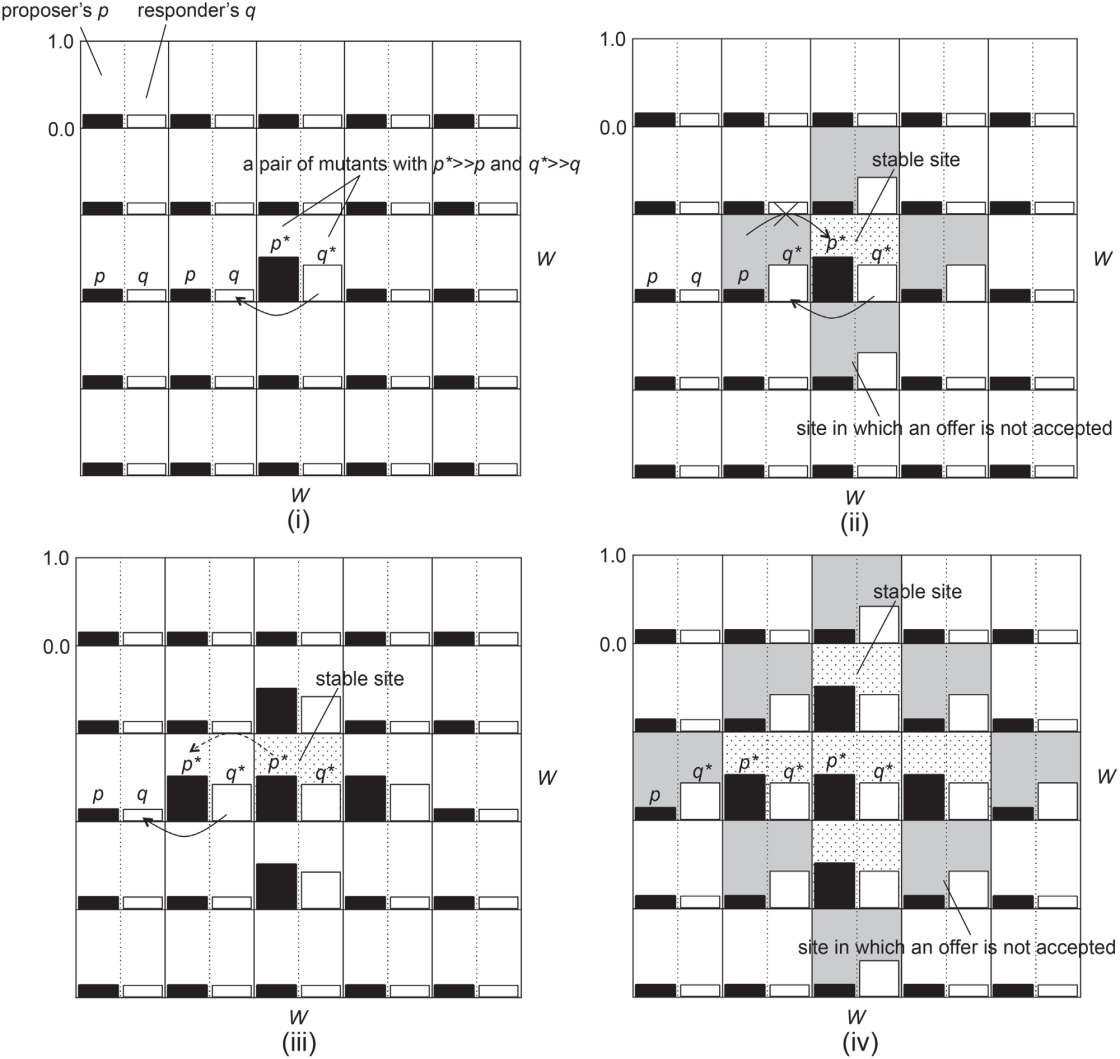
An image of the propagation process of mutant proposer p* and responder q* species with larger *p* and *q* in the case with local interactions between proposer and responder species.

We further investigate the correlated evolution process in the population with the local interactions and the high intensity of selection in detail, as a typical case in which the spatial locality contributed to the emergence of fairness. Fig. 5 is an example evolution of *p* and *q* when *α* = 10^1.0^. We see that a rapid increase and a subsequent decrease in both *p* and *q* occasionally occurred while they also tended to converge to the smaller values around 0.05. This evolution process occurred because a pair of mutant proposer and responder with large *p* and *q* in the same site can gradually invade into the population of rational individuals with small *p* and *q* through the following processes, as illustrated in Fig. 6.

Let us assume that a pair of individuals with much larger p* ≫ *p* and q* ≫ *q* appeared due to mutations or stochastic effects of evolution process in the population of rational individuals with small *p* and *q* that satisfy *p* ≳ *q* (Fig. 6 (i)). In this situation, the mutant responder can obtain much larger fitness than that of the rational responders because of the larger amount of resource proposed by its opponent. This allows the mutant responder to invade into its neighboring sites with the high probability (Fig. 6 (i)). On the other hand, the mutant proposer obtains less fitness than its neighbors’ but there is a certain probability for it to survive because the difference in the fitness between it and its neighbors’ is not so large. Once such a situation occurs (Fig. 6 (ii)), the original pair of mutants tend to survive stably. This is because, in the neighboring sites, the offer from the rational proposer with *p* is not accepted by the mutant responder with q*, which makes the rational proposer *p* impossible to invade into the original site. On the other hand, the mutant proposer with p* can sometimes invade into the neighboring site even when it is not the best neighbor. This is because less adaptive individuals still have small chances to invade into the neighboring sites if the intensity of selection is high (e.g., *α* = 10^1.0^) but not too high to be equivalent to choosing the best (*α* → ∞) completely. Such p* tended to exist for a while because its offer is accepted (Fig. 6 (iii)). In addition, even when the intensity of selection was much higher, an appearance of another mutant proposer who had larger *p* could also bring about the similar situations. This further allows the mutant responder q* to invade into the next neighboring site. As a result, the stable cluster of individuals with larger p* and q* grows (Fig. 6 (iv)).

However, after the whole population is occupied by these fair mutants, there is no such a benefit for larger *p* and *q* because the smaller *q* can accept any offers, which also makes *p* small. Thus, both *p* and *q* gradually decrease and converge to the small values eventually.

It should be noted that such a dynamic process emerged due to the mutual benefit of high fairness for both proposer and responder species in future generations. For the proposer species, the higher fairness is costly in that they obtains less fitness even if its offer is accepted. However, this cost brings about their future benefit in that the fair responder invaded into the neighboring sites can reject the offers from their less fair opponents. As for the fair responder species, keeping the high acceptance threshold is also costly because there is no direct benefit to reject any offer from the proposers. However, this cost enables the proposer species who can bring a better proposal to survive in future generations. In these senses, we can say that fair proposer and responder species pay an immediate cost for their future benefit to survive during the expansion process of their cluster. However, this benefit diminishes when their cluster occupied the whole population.

## Conclusions

We discussed effects of the spatially local interactions and the intensity of selection on the emergence of fair relationship between proposer and responder species in the ultimatum game. We found that the lower intensity of selection favored fair strategies in general, and there were no significant differences in the evolution of fairness between the cases with local and global interactions when the intensity of selection was low. However, as the intensity of selection became higher, the spatially local interactions contributed to the evolution of fairer strategies than the global interactions despite that the fair strategies became more difficult to evolve. This effect of spatially local interactions is expected to be due to the mutual benefit of fairness for both proposer and responder species in future generations, which brought about a dynamic evolution process of fairness.

We have not found real biological cases that directly correspond to a dynamic coevolution process of fairness under the high intensity of selection yet. However, we expect that such a process might have bootstrapped the evolution of a more stable mutualism, we see nowadays, even under the severe condition.

The spatially local interactions are ubiquitous in plant species and expected to have affected the coevolution of the plant and other species including insects. In the pollination-seed consumption mutualisms explained in Introduction [10], the benefit from interactions are obligatory in that it is necessary for their reproductions, implying that the intensity of selection could be strong. Our results imply that, under such a condition, the spatially local interactions can contribute to the emergence of fair strategies among coevolving species.

Future work includes analyzing effects of different topologies of interactions on the coevolution of strategies.
